# The natural agent rhein induces β‐catenin degradation and tumour growth arrest

**DOI:** 10.1111/jcmm.13346

**Published:** 2017-10-11

**Authors:** Shu Liu, Jiao Wang, Ting Shao, Peiying Song, Qingbin Kong, Hui Hua, Ting Luo, Yangfu Jiang

**Affiliations:** ^1^ State Key Laboratory of Biotherapy Section of Oncogene Cancer Center West China Hospital Sichuan University Chengdu China; ^2^ School of Basic Medicine Chengdu University of Traditional Chinese Medicine Chengdu China; ^3^ Laboratory of Stem Cell Biology West China Hospital Sichuan University Chengdu China; ^4^ Cancer Center West China Hospital Chengdu China

**Keywords:** Natural product, rhein, β‐catenin, cancer therapy

## Abstract

The natural agent rhein is an ananthraquinone derivative of rhubarb, which has anticancer effects. To determine the mechanisms underlying the anticancer effects of rhein, we detected the effect of rhein on several oncoproteins. Here, we show that rhein induces β‐catenin degradation in both hepatoma cell HepG2 and cervical cancer cell Hela. Treatment of HepG2 and Hela cells with rhein shortens the half‐life of β‐catenin. The proteasome inhibitor MG132 blunts the downregulation of β‐catenin by rhein. The induction of β‐catenin degradation by rhein is dependent on GSK3 but independent of Akt. Treatment of HepG2 and Hela cells with GSK3 inhibitor or GSK3β knockdown abrogates the effect of rhein on β‐catenin. GSK3β knockdown compromises the inhibition of HepG2 and Hela cell growth by rhein. Furthermore, rhein dose not downregulate β‐catenin mutant that is deficient of phosphorylation at multiple residues including Ser33, Ser37, Thr41 and Ser45. Moreover, rhein induces cell cycle arrest at S phase in both HepG2 and Hela cells. Intraperitoneal administration of rhein suppresses tumour cells proliferation and tumour growth in HepG2 xenografts model. Finally, the levels of β‐catenin are reduced in rhein‐treated tumours. These data demonstrate that rhein can induce β‐catenin degradation and inhibit tumour growth.

## Introduction

The natural agent rhein (4,5‐dihydroxyanthraquinone‐2‐carboxylicacid) is ananthraquinone derivative of rhubarb. Previous studies have demonstrated that rhein and its derivatives have anti‐cancer effects on a variety of human tumours, such as breast cancer 1, 2, hepatocellular carcinoma 3, 4, nasopharyngeal carcinoma 5, 6, 7, cervical cancer 7, 8, 9, 10 and tongue squamous cancer 11. The mechanisms underlying the anti‐cancer effects of rhein may be complex. First, rhein may suppress cancer cell proliferation by inhibiting mitogen‐activated protein kinase and phosphatidylinositol‐4,5‐bisphosphonate 3‐kinase (PI3K)/Akt signalling 12, 13, 14, 15. Second, rhein may induce apoptosis by upregulating Bim expression and caspase activation 16. Third, rhein can inhibit angiogenesis through downregulating hypoxia‐inducible factor‐1 alpha (HIF1α) and vascular endothelial growth factor 15. In addition, both nuclear factor‐kappa B (NF‐κB) and cyclooxygenase 2 (COX‐2), two inflammation‐related factors, are targeted by rhein 1, 15, 17, 18, 19. Other targets of rhein include epidermal growth factor (EGFR) and HER‐2 1, 15.

Similar to growth factors that bind to their receptors and stimulate cellular proliferation, the secreted Wnt proteins bind to the frizzled receptors and LRP5/6 co‐receptors and then initiate a complex signalling cascade to regulate cell proliferation and differentiation 20. β‐catenin is a critical mediator of Wnt signalling. Wnt/β‐catenin signalling plays a vital role in development and growth. Abnormality in Wnt signalling is causatively associated with various diseases, including cancer 21. Oncogenic mutation of β‐catenin has been implicated in colorectal tumorigenesis 22. Overexpression of β‐catenin, a hallmark of activated canonical Wnt pathway, has been observed in a variety of human tumours including breast cancer, lung cancer, ovarian carcinoma, glioma, cervical cancer and colon cancer 23. Oncogenic SATB1 positively regulates β‐catenin expression 24. In addition, the stabilization of β‐catenin protein is a critical mechanism underlying the aberrant expression of β‐catenin. In the classical β‐catenin destruction complex, glycogen synthase kinase‐3β (GSK‐3β) phosphorylates β‐catenin at its N‐terminus, and phosphorylated β‐catenin is then recognized by F‐box β‐transducin repeat‐containing protein (BTRC), a component of the ubiquitin ligase complex, resulting in the degradation of β‐catenin 25.

As a transcriptional activator, β‐catenin directly interacts with transcription factors such as LEF‐1 and regulates transcription of target genes such as c‐myc and cyclin D1. Cyclin D1 encodes the regulatory subunit of a holoenzyme that phosphorylates and inactivates the retinoblastoma protein and promotes cell cycle progression 26. Overexpression of cyclin D1 is known to correlate with tumour onset and progression 26. Activation of Wnt/β‐catenin signalling pathway may play important roles in tumour development. The nodal points of Wnt/β‐catenin signalling pathway may be promising targets for cancer drug discovery 20. Here, we report that rhein promotes β‐catenin degradation in HepG2 and Hela cells. Treatment of HepG2 and Hela cells with rhein leads to cell cycle arrest and slows down cell proliferation. Treatment of tumour xenografts with rhein suppresses β‐catenin expression and tumour growth.

## Materials and methods

### Reagents and antibodies

Rhein was purchased from MUST Biotech (Chengdu, China). LiCl was purchased from Sigma‐Aldrich (St. Louis, MO, USA). MG132 was from Merk Millipore Corporation (Darmstadt, Germany). Cycloheximide (CHX) was from Beyotime Biotechnology (Jiangsu, China). Anti‐β‐catenin, anti‐c‐Myc, anti‐PCNA, anti‐phosphorylated GSK‐3β (Ser9) antibodies were from Epitomics (Burlingame, CA, USA). Anti‐GSK3 antibody was from Signal way Antibody LLC (College Park, MD, USA). Anti‐cyclin‐D1 antibody was from Proteintech (Rosemont, IL, USA). The plasmid for phosphorylation‐deficient mutant of β‐catenin (S33A, S37A, T41A, S45A) was from Addgene (Cambridge, MA, USA).

### Cell culture

Hepatoma cancer cell line HepG2 and cervical cancer cell line Hela were obtained from Cell Lines Bank, Chinese Academy of Science (Shanghai, China) and grown in DMEM supplemented with 10% new born calf serum (Thermo Fisher Scientific, Waltham, MA USA). The cells were incubated at 37°C in a humidified atmosphere of 5% CO_2_.

### RNA interference

All siRNAs were custom‐synthesized products of Ribobio Co., Ltd. (Guangzhou, China). Target sequences for GSK‐3β knockdown are 5′‐GAUCUGUCUUGAAGGAGAA‐3′. Target sequences for Axin2 and BTRC knockdown are 5′‐GAGUAGCCAAAGCGAUCUA‐3′ and 5′‐CACAUAAACUCGUAUCUUA‐3′, respectively. Subconfluent proliferating cells were incubated with 50 nM siRNA or 4 μg plasmid in 2 ml of OPTI‐MEM^®^I Reduced Serum Medium containing Lipofectamine 2000 (Thermo Fisher Scientific). Forty‐eight hours after transfections, cells were harvested with cold lysis buffer (50 mM Tris‐HCl [pH 7.4], 0.25% sodium deoxycholate, 150 mM NaCl, 1% nonidet P‐40 [NP‐40], 1 mM EDTA, 1 mM NaF, 1 mM Na3V4, 1 mM phenylmethylsulfonyl fluoride) and stored in −80°C.

### Plasmids construction

Plasmids for wild‐type Akt1 (WT‐AKT1) and constitutively active Akt1 (CA‐AKT1) were purchased from Addgene. The cDNAs were subcloned into pcDNA3.1(+) (Thermo Fisher Scientific) with four copies of hemaglutinin (HA) tags. The cDNAs encoding Jade1 lacking two PHD domains and Siah1 lacking the ring domain were amplified by PCR from HepG2 cells randomly primed cDNA libraries and then subcloned into pcDNA3.1‐HA.

### Western blot analysis

Western blotting was carried out following standard procedures. In brief, after being quantified by BCA protein assay (Thermo Fisher Scientific), aliquots of 30 μg proteins were loaded into 10% Tris–HCl polyacrylamide gels and transferred to PVDF membranes (Merk Millipore Corporation). Membranes were incubated with primary antibodies and appropriate HRP‐secondary antibodies. Detection was performed with chemiluminescent agents. Images were gathered by the Alpha Innotech FluorChem imaging system (Alpha Innotech Corp., San Leandro, CA, USA).

### Real‐time reverse transcription‐PCR

Total RNA were extracted from cultured cells using Trizol reagent (Thermo Fisher Scientific) according to the manufacturer's protocol. First strand cDNAs were synthesized using the MMLV reverse transcriptase and oligo (dT) primers. β‐catenin and GAPDH were amplified by real‐time PCR using the SYBR^®^ Select Master Mix (Thermo Fisher Scientific). The primer sequences for human β‐catenin were 5′‐ GTGCTATCTGTCTGCTCTAGTA ‐3′ (forward) and 5′‐CTTCCTGTTTAGTTGCAGCATC ‐3′ (reverse). The primer sequences for GAPDH were 5′‐AATCGCATCATCATAACCTG‐3′ (forward) and 5′‐CATCCTGCCCATCATACTC‐3′ (reverse). Relative quantification with the comparative threshold cycle (Ct) was performed using the Ct method. The amount of β‐catenin gene normalized to the endogenous reference gene (GAPDH) is given by 2‐ΔCt, where ΔCt is Ct (β‐catenin) ‐ Ct (GAPDH).

### CCK‐8 assay

The cells were seeded in 96‐well plates and allowed to attach and grow for 24 hrs. Then, the cells were treated with various concentrations of rhein for 48 hrs. Cell viability was assessed by incubating cells with CCK‐8 (Cell Counting Kit‐8) reagents (Dojindo Laboratory Co., Ltd., Kumamoto, Japan) for 1–2 hrs and measuring the absorbance at 450 nm with a microplate reader (Bio‐Rad, Hercules, CA, USA). The IC50 values were calculated using the GraphPad Prism Software (Version 6.01) (La Jolla, CA, USA).

### Cell‐Light™EdU assay

The cells were seeded in 96‐well plates at 5000 cells per well. Twenty‐four hours later, the cells were treated with rhein in different concentrations. After 48 hrs, cell proliferation was measured by Cell‐Light™ EdU Apollo 567 *In Vitro* Kit (RiboBio Co. Ltd), according to the manufacturer's protocol. Images were captured using a fluorescence microscope.

### *In vitro* kinase assay

The kinase assays were conducted as described previously 27. Briefly, one mg of total proteins was immunoprecipitated with 3 μg of indicated antibodies for 90 min. at 4°C. Target proteins were collected by incubation with protein G Sepharose beads for 60 min. at 4°C, followed by washing three times with cold lysis buffer and once with cold kinase buffer (25 mM Hepes pH 7.5, 100 mM potassium acetate, 1 mM MgCl_2_). Then, the beads–proteins complexes were used for kinase reaction. The peptide RRAAEELDSRAGpSPQL was used as the substrate of GSK3β. The kinase activity was monitored by ELISA analysis of peptide substrate phosphorylation.

For ELISA, each well of the polystyrene plate was coated with 50 μl coating buffer (15 mM Na2CO3, 35 mM NaHCO3, PH 7.4) containing 2 μg of polypeptide overnight at 4°C, followed by washing three times with PBST (NaCl 8 g/l, KCl 0.2 g/l, Na2HPO4 1.44 g/l, KH2PO4 0.24 g/l, 0.1% tween‐20 (v/v), PH 7.4) and one time with the kinase buffer. The plate was incubated in a final volume of 50 μl/well at 37°C for 1 hr in kinase buffer containing 500 μM ATP with or without kinase. After the reaction, the plate was washed with PBST and then incubated with appropriate primary antibodies and secondary antibodies. After washing the plate with PBST for 8–10 times, the plate was incubated with TMB solution (Na_2_HPO_4_ 14.6 g/l, citric acid 9.3 g/l, TMB (tetramethyl benzidine) 0.5 g/l, H_2_O_2_ 0.025‰ (v/v), PH 5.0.) for 30 min. at 37°C. Then, the TMB reaction was stopped by adding 50 μl 10% H2SO4 per well, followed by detecting absorbance at 450 nm by microplate reader.

### Flow cytometry analysis

The cells were seeded into 6‐well plates at a concentration of 5 × 10^5^/well and allowed to attach overnight, then treated with rhein (40 μM) for 48 hrs and harvested. For cell cycle analysis, the cells were fixed in 70% ice‐cold ethanol at 4°C overnight. The cells were then washed with ice‐cold PBS and treated with RNase for 20 min. before stained with PI (100 μg/ml) at room temperature. The samples were analysed by a FACSCalibur Flow Cytometer (BD Biosciences, San Jose, CA, USA). Three independent experiments were performed.

### Cancer xenograft model

Female nude mice (BALB/c‐nu) were purchased from the Experimental Animal Center of Sichuan University. Five‐week‐old mice (*n* = 20) were inoculated subcutaneously with 5 × 10^6^ HepG2 cells in 100 μl PBS. One week later, the mice were randomly divided into two groups (*n* = 10 mice/group) and were given intraperitoneal (i.p.) injection of rhein (100 mg/kg/0.2 ml, once a day) or same volume of vehicle (1M Na_2_CO_3_:1M NaHCO_3_ = 4:6, 20% PEG300, pH 7.5). Tumour width (W) and length (L) were measured every 3 days by callipers. The tumour volume (Tv) was calculated according to the formula: Tv = 0.52 × L × W^2^. After 3 weeks of treatment, the mice were killed, and the tumours were removed, weighed and subjected to further experiments. All studies involving mice were approved by the Animal Care and Use Committee of West China Hospital. All experiments were carried out in accordance with the approved guidelines.

### Immunohistochemistry

Tissues were formalin‐fixed and paraffin‐embedded, and sections were consecutively cut into 3–4 μm thickness for immunohistochemical analysis. The slides were heated in 60°C for 2 hrs and subsequently deparaffinized with dimethylbenzene, rehydrated through a series of decreasing concentrations of ethanol, and incubated in 3% H_2_O_2_ for 10 min. in dark at room temperature to quench the endogenous peroxidase activity. The immunohistochemical staining was performed by using the SP‐9001 kit (Zhong Shan Jin Qiao, Beijing, China). Tissue sections were subjected to epitope recovering in citrate buffer (pH 6.0) at 99°C for 5 min. Slides were then washed in PBS and blocked by goat serum for 20 min. at room temperature. Sections were incubated overnight at 4°C with the following primary antibodies: rabbit anti‐PCNA, anti‐β‐catenin and anti‐cyclinD1. After washing in PBST, slides were incubated with biotin‐labelled secondary antibodies at 37°C for 40 min. Sections were then washed in PBST and incubated with streptavidin‐HRP at 37°C for 30 min. After washing, sections were covered with freshly prepared DAB solution and immediately washed under tap water after colour development. Sections were then counterstained with haematoxylin. Analysis of immunohistochemical staining was performed by Image‐Pro Plus (version 6.0), the measurement parameters were the unit optical density values (density mean), the mean density was calculated, and the intensity was averaged from ten fields of view.

### Statistical analyses

Statistical differences analysed by anova and by unpaired two‐tailed Student's *t*‐test. *P* values less than 0.05 are statistically significant.

## Results

### Rhein suppresses HepG2 and Hela cells proliferation

**Figure 1:**
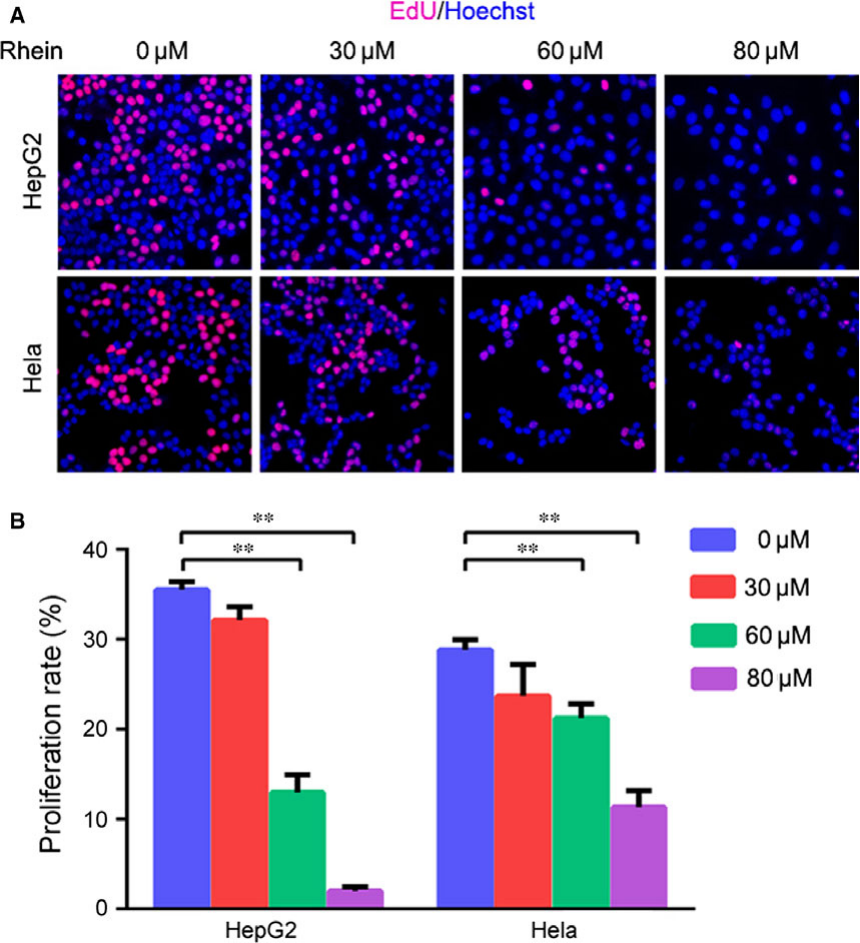
Rhein suppresses the proliferation of HepG2 and Hela cells. (**A**) HepG2 and Hela cells were treated with increasing dose of rhein for 48 hrs, followed by labelling with EdU and Hoechst 33342. (**B**) The proliferation rate was plotted. Values represent mean ± S.D. **P < 0.01.

Previously, we reported that rhein induced cancer cell apoptosis through FOXO3‐ and Bim‐dependent mechanisms 16. To detect the effescts of rhein on cancer cells proliferation, we treated HepG2 and Hela cells with increasing doses of rhein for 48 hrs, followed by EdU and Hoechst 33342 labelling. The positivity of EdU‐labelled cells was significantly decreased by treatment with 60 μM or more rhein in both HepG2 and Hela cells, while the sensitivity to rhein varied between these two cell lines (Fig. 1). Our previous study demonstrated that the mean IC50 value of rhein was 34.5 μM for HepG2 cells 16. To determine the IC50 value for Hela cells, we treated Hela cells with increasing doses of rhein for 24–96 hrs, followed by CCK‐8 assay. The IC50 of rhein at 72 hrs was 53.97 μM for Hela cells.

### Rhein promotes proteasomal degradation of β‐catenin

**Figure 2:**
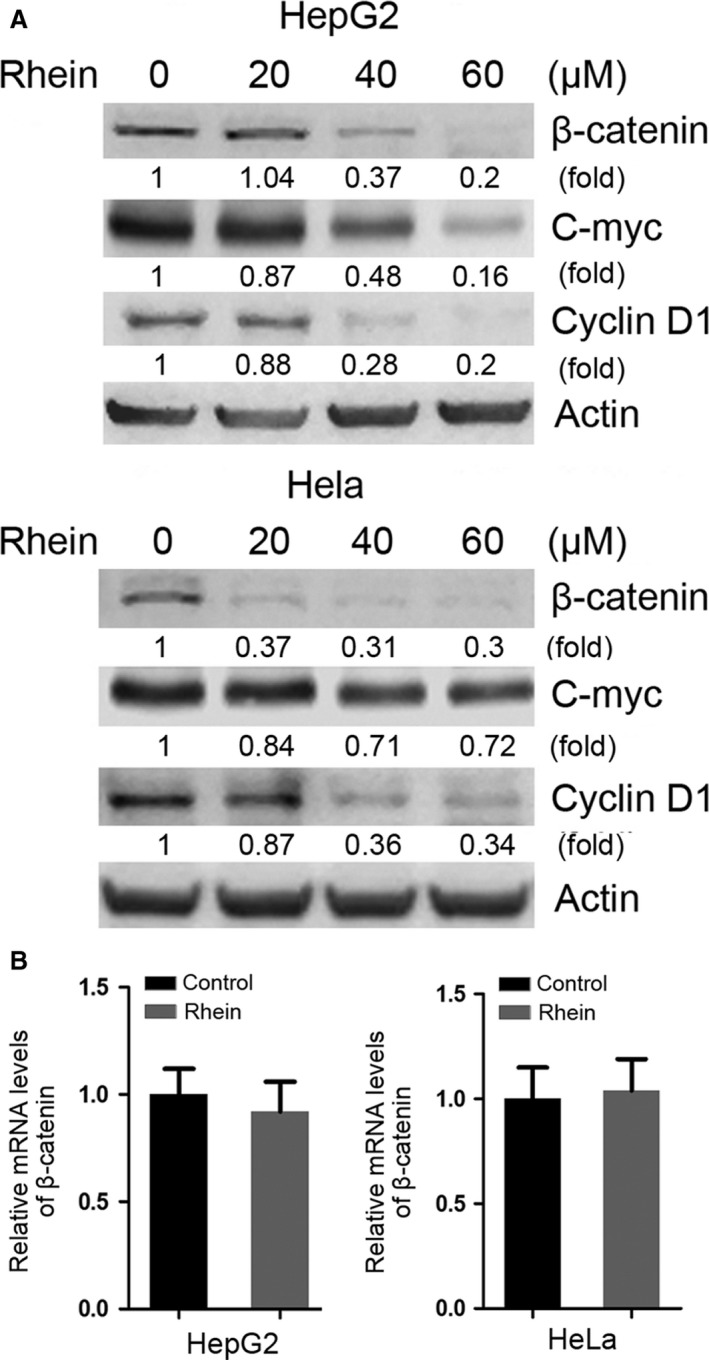
Rhein decreases the protein level of β‐catenin without changing its mRNA level. (**A**) HepG2 and Hela cells were treated with rhein in increasing dose indicated for 48 hrs followed by Western blot analysis of β‐catenin, c‐myc and cyclin D1. (**B**) HepG2 and Hela cells were treated with 60 μM rhein for 48 hrs, followed by real‐time RT‐PCR analysis of β‐catenin transcription. The relative mRNA levels of β‐catenin were plotted. Values represent means ± S.D. (*n* = 3). The mean level of β‐catenin mRNA in rhein‐untreated group was set as 1.

**Figure 3:**
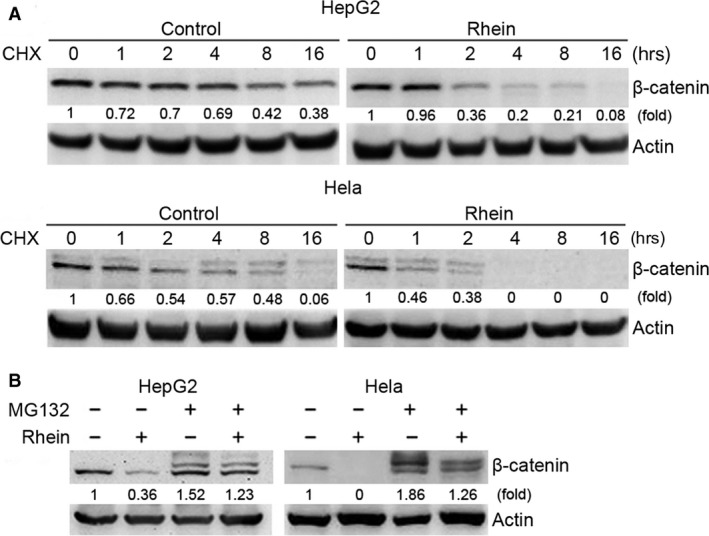
Rhein promotes proteasomal degradation of β‐catenin. (**A**) HepG2 and Hela cells were treated with a protein translation inhibitor, CHX (100 μg/ml), and with or without rhein (100 μM). At the indicated time‐point, cells were collected followed by Western blot analysis of β‐catenin. (**B**) HepG2 and Hela cells were treated with or without rhein (60 μM) and proteasome inhibitor MG132 (5 μM) for 48 hrs, followed by Western blot analysis of β‐catenin.

To investigate the mechanism by which rhein inhibits cell proliferation, we examined the effects of rhein on the expression of several proliferation‐related proteins, including β‐catenin, p53 and K‐ras. Among them, the levels of β‐catenin were reduced by treatment with rhein in both HepG2 and Hela cells (Fig. 2A). Meanwhile, the levels of c‐myc and cyclin D1, two targets of β‐catenin, were decreased. However, rhein did not have significant effect on the mRNA level of β‐catenin (Fig. 2B), suggesting that rhein downregulated β‐catenin through post‐transcriptional mechanism rather than repressing its gene expression. We then determined the effect of rhein on the half‐life of β‐catenin protein in HepG2 and Hela cells. As shown in Figure 3A, rhein dramatically decreased the half‐life of β‐catenin in both HepG2 and Hela cells. Given that β‐catenin is a target for proteasomal degradation, we determined whether the proteasome inhibitor MG132 could antagonize the downregulation of β‐catenin by rhein. MG132 abolished the effect of rhein on β‐catenin (Fig. 3B), indicating that rhein induces proteasomal degradation of β‐catenin.

### The induction of β‐catenin degradation by rhein is dependent on GSK3 but independent of Akt

**Figure 4:**
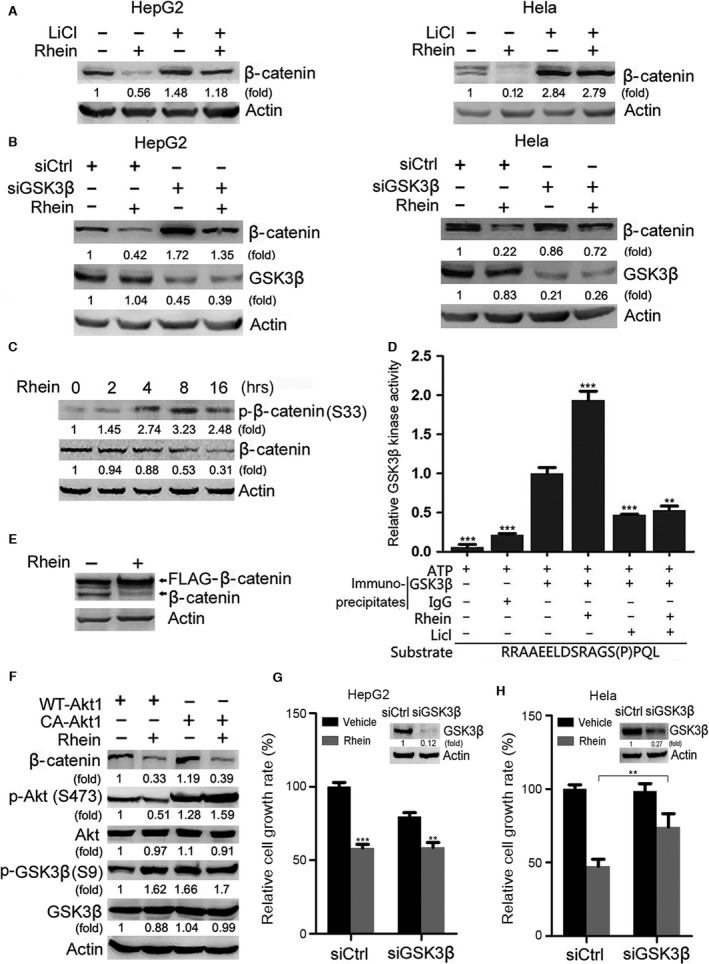
GSK‐3β is involved in rhein‐induced degradation of β‐catenin and inhibition of cell growth. (**A**) HepG2 and Hela cells were treated with or without LiCl (10 mM) and rhein (60 μM) for 48 hrs, followed by Western blot analysis of β‐catenin. (**B**) HepG2 and Hela cells were transfected with GSK‐3β siRNA. Twenty‐four hours after transfection, the cells were treated with or without rhein (60 μM) for 48 hrs, followed by Western blot analysis of β‐catenin and GSK‐3β. (**C**) HepG2 cells were treated with or without rhein (100 μM) for indicated periods, followed by Western blot analysis. (**D**) *In vitro* kinase assays of immunoprecipitated GSK3β. HepG2 cells were treated with or without 100 μM rhein for 24 hrs, followed by immunoprecipitation with anti‐GSK3β antibody or normal IgG. The immunoprecipitates were subjected to *in vitro* kinase assays in the presence of a peptide substrate. The relative kinase activity of GSK3β was plotted. **P < 0.01; ***P < 0.001, compared to the kinase activity of GSK3β immunoprecipitates from rhein‐untreated cells, which was set as 1. (**E**) HepG2 cells were transduced with FLAG‐tagged phosphorylation‐deficient mutant of β‐catenin (S33A, S37A, T41A, S45A), followed by treatment with or without rhein (100 μM) for 24 hrs. The endogenous β‐catenin and FLAG‐tagged phosphorylation‐deficient mutant of β‐catenin were detected by Western blotting. (**F**) HepG2 cells were transduced with wild‐type Akt1 (WT‐Akt1) or constitutively active Akt1 (CA‐Akt1), followed by treatment with or without rhein (100 μM) for 24 hrs. The effects of Akt and rhein on β‐catenin expression, Akt and GSK3β phosphorylation were detected by Western blotting. (**G**) HepG2 cells were transduced with negative control siRNA (siCtrl) or GSK3β siRNA (siGSK3β). Twenty‐four hours later, the cells were treated with or without rhein (60 μM) for another 24 hrs, followed by CCK8 assays. The relative cell growth rate was plotted. In parallel, the efficiency of GSK3β knockdown was detected by Western blotting. ***P < 0.001; **P < 0.01, compared with siControl. (H) Hela cells were transduced with siCtrl or siGSK3β. Twenty‐four hours later, the cells were treated with or without rhein (60 μM) for another 48 hrs, followed by CCK8 assays. The relative cell growth rate was plotted. In parallel, the efficiency of GSK3β knockdown was detected by Western blotting. **P < 0.01.

Given that the phosphorylation of β‐catenin by GSK‐3β plays a key role in proteasomal degradation of β‐catenin, we examined whether GSK3β was involved in the induction of β‐catenin degradation by rhein. Western blot analysis showed that LiCl, a GSK3 inhibitor, abrogated the induction of β‐catenin degradation by rhein (Fig. 4A). In addition, GSK3β knockdown blunted the induction of β‐catenin degradation by rhein (Fig. 4B), suggesting that the induction of β‐catenin degradation by rhein is dependent on GSK3 protein.

β‐catenin can be phosphorylated by GSK3β at multiple residues including Ser33. Treatment of HepG2 cells with rhein resulted in increased phosphorylation of β‐catenin at Ser33, suggesting that rhein may upregulate GSK3β activity (Fig. 4C). *In vitro* kinase assays of immunoprecipitated GSK3β demonstrated that rhein did upregulate the kinase activity of GSK3β (Fig. 4D). Moreover, rhein failed to downregulate phosphrylation‐deficient mutant (S33/37A, T41A, S45A) of β‐catenin (Fig. 4E). GSK3 may be phosphorylated and inactivated by Akt. While rhein inhibited Akt phosphorylation, it still upregulated GSK3β phosphorylation at Ser9, a target site of Akt and other kinases (Fig. 4F). These data suggest that GSK3β may be active even if Ser9 is phosphorylated. Furthermore, overexpression of constitutively active Akt did not rescue β‐catenin levels in rhein‐treated cells, suggesting that the induction of β‐catenin degradation by rhein did not result from Akt inhibition (Fig. 4F). Moreover, GSK3β‐knockdown inhibited HepG2 cell growth, but had no effect on Hela cell growth (Fig. 4G and H). In both HepG2 and Hela cells, the extent to which rhein inhibited growth in GSK3β‐knockdown cells was significantly less than that in non‐knockdown cells (Fig. 4G and H), indicating that GSK3β contributes in part to the inhibition of cell growth by rhein.

**Figure 5:**
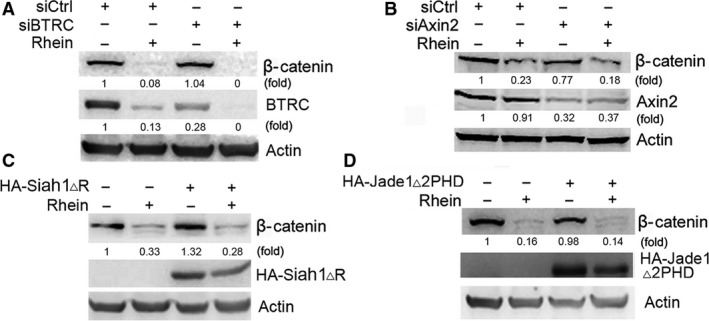
BTRC, Siah1 and Jade1 are not involved in the induction of β‐catenin degradation by rhein. (**A**) HepG2 cells were transduced with negative control siRNA (siCtrl) or BTRC siRNA (siBTRC), followed by treatment with or without rhein (100 μM) for 24 hrs. The levels of β‐catenin and BTRC were detected by Western blotting. (**B**) HepG2 cells were transduced with negative control siRNA (siCtrl) or axin2 siRNA (siAxin2), followed by treatment with or without rhein (100 μM) for 24 hrs. The levels of β‐catenin and axin2 were detected by Western blotting. (**C**) HepG2 cells were transduced with empty vector or HA‐Siah1▵R, followed by treatment with or without rhein (100 μM) for 24 hrs. The levels of β‐catenin and HA‐Siah1▵R were detected by Western blotting. (**D**) HepG2 cells were transduced with empty vector or HA‐Jade1▵2PHD, followed by treatment with or without rhein (100 μM) for 24 hrs. The levels of β‐catenin and HA‐Jade1▵2PHD were detected by Western blotting.

Previous study reported that BTRC, an E3 ubiquitin ligase and component of β‐catenin destruction complex, couples β‐catenin phosphorylation and degradation 25, 28. However, rhein inhibited BTRC expression (Fig. 5A). BTRC knockdown had no effect on the induction of β‐catenin degradation by rhein (Fig. 5A). In addition, knockdown of Axin2, a component of the β‐catenin destruction complex, had no effect on the induction of β‐catenin degradation by rhein (Fig. 5B). These data indicate that the induction of β‐catenin degradation by rhein may be independent of β‐catenin destruction complex. Moreover, overexpression of dominant‐negative mutants of Jade1 and Siah1, which reportedly induce β‐catenin degradation 29, 30, 31, did not abrogate the induction of β‐catenin degradation by rhein (Fig. 5C and D).

### Rhein induces cell cycle arrest at S phase

**Figure 6:**
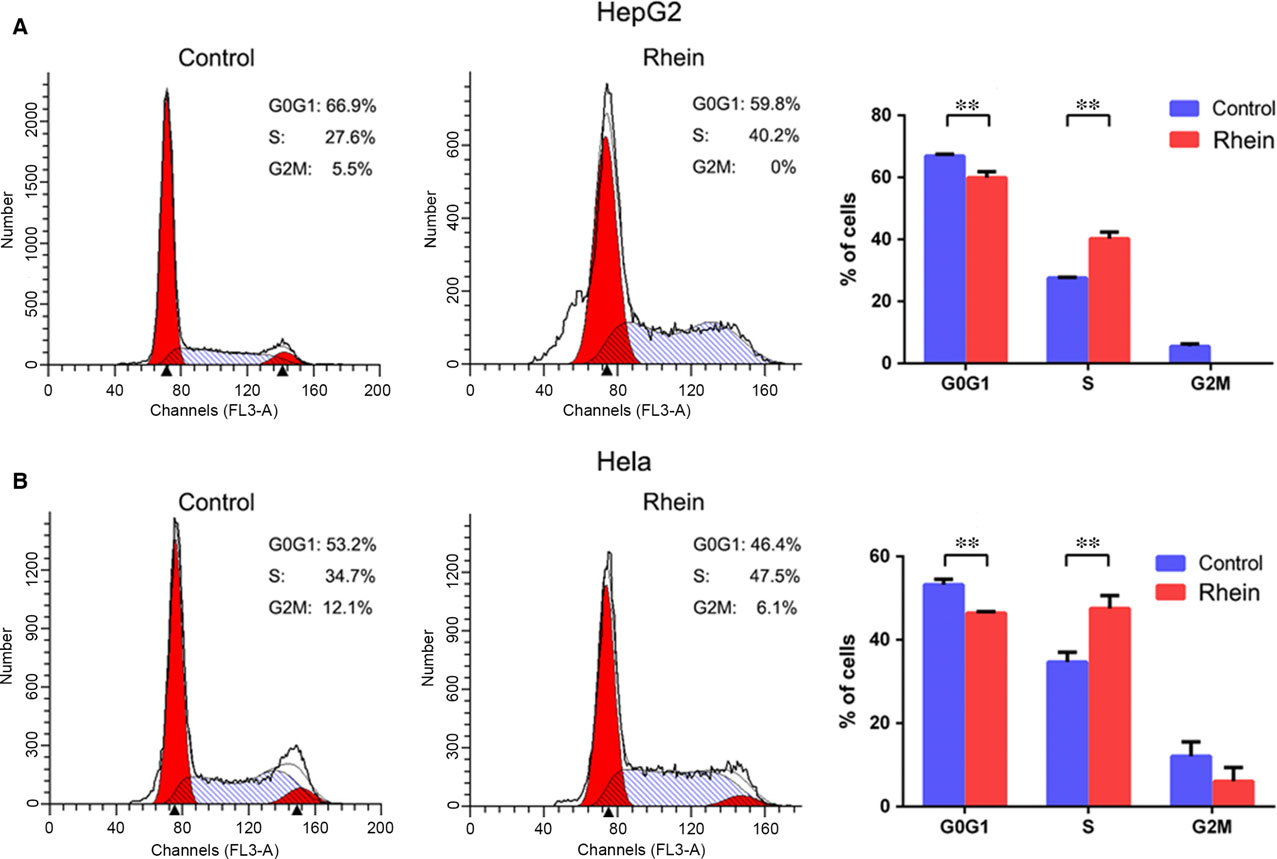
Rhein induces cell cycle arrest. (**A**) HepG2 cells were treated with rhein (40 μM)for 48 hrs and stained with propidium iodide (PI). The proportion of cells in each phases were analysed by flow cytometry. (**B**) Hela cells were treated with rhein (40 μM)for 48 hrs and stained with propidium iodide (PI). The proportion of cells in each phases were analysed by flow cytometry. The mean values from three independent experiments were plotted. *Bars*, SD. **P < 0.01.

The above‐mentioned data demonstrated that rhein reduced β‐catenin and cyclin D1 expression. We then determined the effects of rhein on cell cycle progression. To this end, we treated HepG2 and Hela cells with 40 μM rhein for 48 hrs and analysed the cell cycle distribution by flow cytometry. The results showed that rhein arrested both HepG2 and Hela cells at S‐phase (Fig. 6).

### Rhein inhibits β‐catenin expression and tumour growth *in vivo*

**Figure 7:**
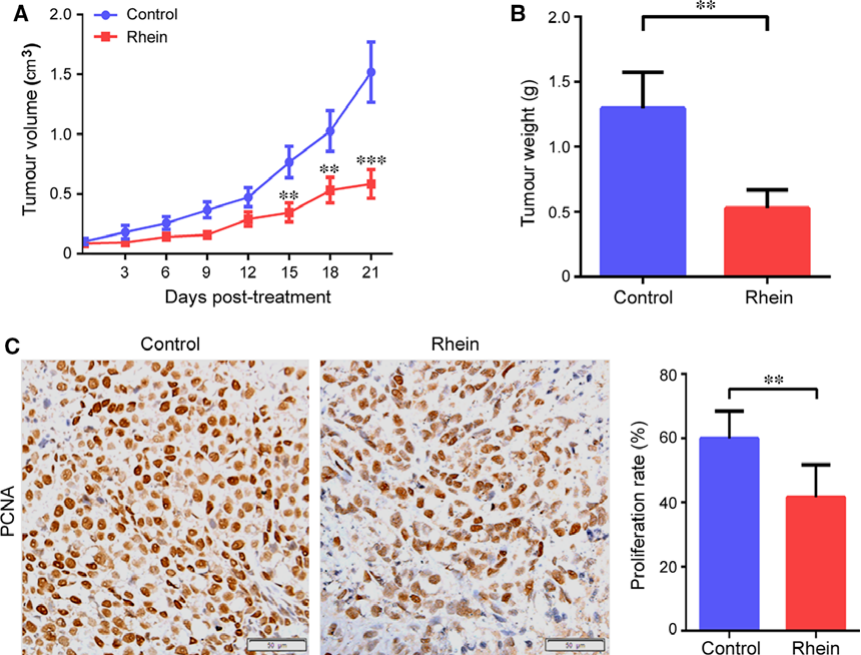
Rhein displays anticancer activity *in vivo*. (**A**) HepG2 cells were injected into Balb/c‐nu mice as described in Materials and methods. When tumours became palpable, the mice were randomly divided into two groups (*n* = 10 mice/group), and were given intraperitoneal (i.p.) injection of rhein (100 mg/kg/0.2 ml, once a day) or same volume of vehicle. Tumour width (W) and length (L) were measured every 3 days by callipers. Tumour growth curve was plotted. *Bars*, SD. **P < 0.01 when comparing the rhein‐treated group with the control. (**B**) The tumours weight was measured. *Bars*, SD. **P < 0.01. (**C**) Representative tumour tissue sections (100× magnification) stained for PCNA by immunohistochemistry. Proliferation rate was assessed in terms of the percentage of the PCNA‐positive cells. The proliferation rate was plotted. *Bars*, SD. **P < 0.01. A representative of two independent experiments was shown.

**Figure 8:**
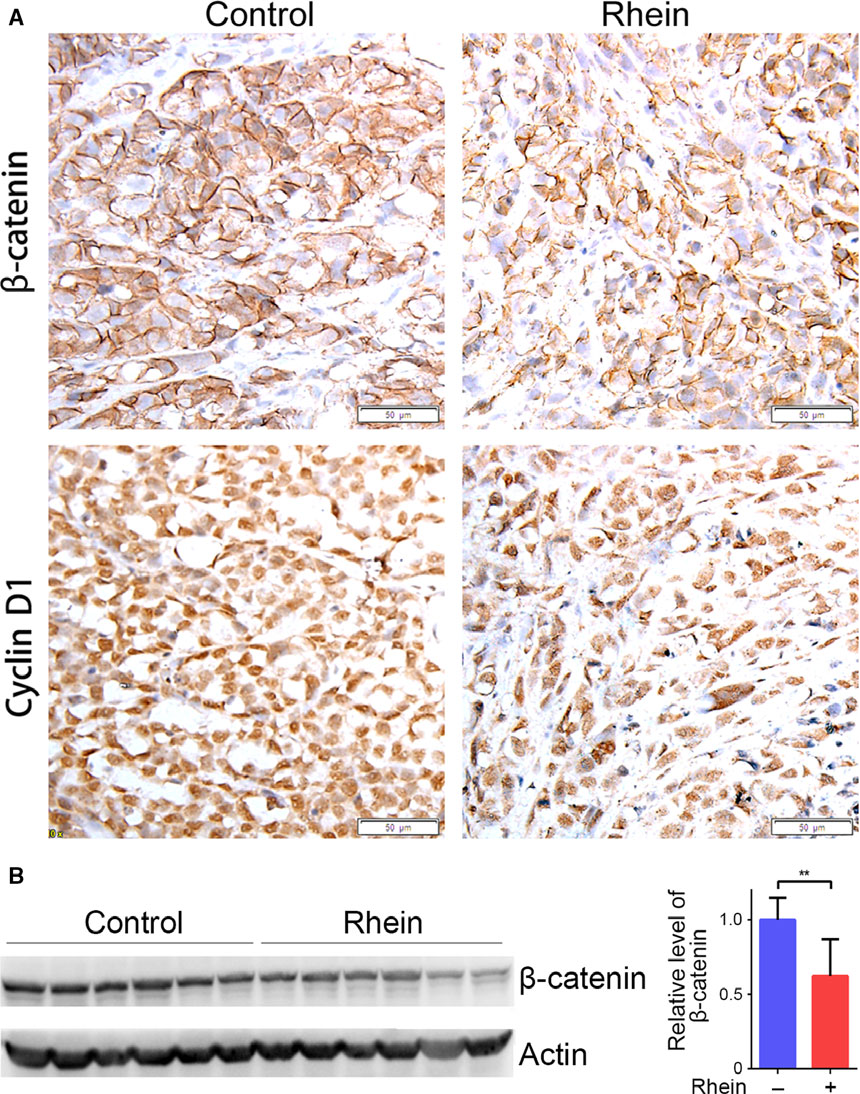
Rhein inhibits β‐catenin expression in tumour xenografts. (**A**) Representative tumour tissue sections (100 × magnification) stained for β‐catenin by immunohistochemistry. (**B**) Tumour lysates were subjected to Western blot analysis of β‐catenin. The blots were subjected to densitometric analysis and relative quantification. **P < 0.01.

To further evaluate the anti‐tumour effects of rhein *in vivo*, we used a HepG2 xenograft nude mouse model. In comparison with the vehicle (control), intraperitoneal administration of rhein significantly inhibited HepG2 tumour growth in nude mice, as reflected in the obvious reduction in tumour size and weight (Fig. 7A and B). Immunohistochemistry (IHC) for PCNA showed decreased proliferation in the rhein‐treated tumours as compared to control (Fig. 7C). In addition, Western blot and immunohistochemical analysis showed that rhein downregulated β‐catenin level in tumour xenografts (Fig. 8). These data demonstrate that rhein can inhibit β‐catenin expression and tumour growth *in vivo*.

## Discussion

Aberrant Wnt/β‐catenin signalling is implicated in the progression of human malignancies. The important downstream targets of β‐catenin/Tcf transactivation complex include SATB1 24, SP5 32, ITF‐2 33. Overexpression of β‐catenin and its downstream targets may promote cancer cell proliferation, invasion, stemness and drug resistance. Hence, β‐catenin is an attractive target for cancer therapeutics 34. There are many strategies to target Wnt/β‐catenin signalling. First, structure‐based development of β‐catenin/TCF4 inhibitors is a direct route for drug discovery. Computational and structure‐based design resulted in the design and synthesis of a series of anthracene‐9, 10‐dione dioxime series of compounds demonstrated potent inhibition of β‐catenin *in vitro* (IC50 < 10 nM, 14, BC‐2059) and strongly inhibited the growth of several cancer cell lines 35. Second, Wnt/β‐catenin signalling can be repressed by inhibition of β‐catenin/TCF4 interaction 36, 37. Stimulating the activity of the β‐catenin destruction complex is another strategy to inhibit β‐catenin. Our current study demonstrates that the natural anthracycline rhein can induce β‐catenin degradation. Thus, inhibition of β‐catenin may represent a novel mechanism underlying the anti‐cancer effects of rhein.

β‐catenin is degraded through proteasomal pathways regulated by GSK3β, p53‐inducible Siah1, etc. 30, 31, 38. In this study, inhibition of GSK3β abrogates rhein‐induced β‐catenin degradation, suggesting that the induction of β‐catenin degradation by rhein is dependent on GSK3. Akt can inactivate GSK3β through Ser9 phosphorylation. While rhein inhibits Akt phosphorylation, it still induces GSK3β phosphorylation at Ser9, suggesting that rhein may activate other kinases to induce GSK3β phosphorylation. Meanwhile, rhein increases the kinase activity of GSK3β and upregulates β‐catenin phosphorylation at GSK3β‐targeted site. Although GSK3β activity may be regulated by Ser9 phosphorylation, GSK3β phosphorylation at Ser9 does not exactly reflect its kinase activity 38, 39. Indeed, there are other mechanisms that may regulate GSK3β activity by counteracting the inhibitory effects of phosphorylation of GSK3β at Ser9 39, 40. The E3 ubiquitin ligase BTRC can trigger β‐catenin degradation 25, 28. However, we find that rhein inhibits BTRC expression. BTRC knockdown fails to rescue rhein‐induced β‐catenin degradation, indicating that BTRC is not required for the induction of β‐catenin degradation by rhein. Besides BTRC, Jade1 is another E3 ligase capable of inducing β‐catenin degradation 29. However, overexpression of a dominant‐negative form of Jade1 did not interfere with rhein‐induced β‐catenin destabilization. It remains to know whether there are other mechanisms underlying β‐catenin degradation.

As a natural compound, rhein has anticancer effects *in vitro* and *in vivo*. Based on our current study and other reports, rhein can inhibit cell proliferation in a variety of cancer cells 2, 10, 12, 41. Besides β‐catenin, other targets of rhein include ERK, p38 MAPK, JNK, PI3K/Akt and FTO 2, 10, 12, 37, 41, 42. One of the targets of β‐catenin is cyclin D1, a key molecule facilitating cell cycle progression 26. Another downstream target of β‐catenin is c‐myc, a proto‐oncogene also involved in cell cycle progression 43. We observed a decreased expression of both cyclin D1 and c‐myc in rhein‐treated HepG2 and Hela cells. Besides β‐catenin, cyclin D1 and c‐myc, rhein may affect cell cycle progression through other targets such as p53 and p21 12, 44, 45. Also, the effect of rhein on cell cycle may be cell‐type specific. Previous studies demonstrate that rhein induces cell‐cycle arrest at G0/G1 and S phase in A549 and BEL‐7402 cells 3, 46, respectively. In the current study, we find that rhein arrests HepG2 and Hela cells at S phase.

Although rhein has anticancer effects *in vitro*, the poor solubility of rhein in water is a disadvantage for the administration *in vivo*. We find that the solubility of rhein can be improved by dissolving it in sodium carbonate buffer and heating. This formula has comparable effects to rhein dissolved in DMSO *in vitro*. In addition, i.p. injection of dissolved rhein in sodium carbonate buffer results in significant anti‐cancer effects *in vivo*. The lapactic effect is an adverse effect during treatment with rhein, which may be controlled by symptomatic therapy. Light salt water is a kind of replenisher to relieve dehydration. Rhein may be a candidate anti‐cancer drug or a lead compound to develop more powerful drugs.

## Conflict of interest

The authors confirm that there are no conflicts of interest.
