# Correction to ‘The Natural Agent Rhein Induces β‐catenin Degradation and Tumour Growth Arrest’

**DOI:** 10.1111/jcmm.71075

**Published:** 2026-02-24

S. Liu, J. Wang, T. Shao, et al., “The Natural Agent Rhein Induces β‐Catenin Degradation and Tumour Growth Arrest,” *Journal of Cellular and Molecular Medicine* 22 (2018): 589–599. https://doi.org/10.1111/jcmm.13346.

In Shu Liu et al., the actin blot of HepG2 cell line in Figure 4A was a duplicate of the actin blot of HepG2 in Figure 3B. The correct Figure 4A is shown below. The authors confirm all results and conclusions of this article remain unchanged.

**FIGURE 4A:**
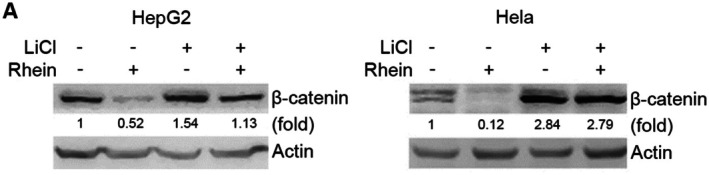
GSK‐3β is necessary for rhein‐induced degradation of β‐catenin. (A) HepG2 and Hela cells were treated with or without LiCl (10 mM) and rhein (60 μM) for 48 h, followed by western blot analysis of indicated proteins.

We sincerely apologise for this error.

